# Epidemiology and treatment outcomes of diabetic retinopathy in a diabetic population from Cameroon

**DOI:** 10.1186/1471-2415-14-19

**Published:** 2014-02-24

**Authors:** Ahmadou M Jingi, Jean Jacques N Noubiap, Augustin Ellong, Jean Joel R Bigna, Côme Ebana Mvogo

**Affiliations:** 1 Department of Internal Medicine and Specialties, Faculty of Medicine and Biomedical Sciences, University of Yaoundé I, Yaoundé, Cameroon; 2 Internal Medicine Unit, Edéa Regional Hospital, PO Box 100, Edéa, Cameroon; 3 Department of ophthalmology, Faculty of Medicine and Biomedical Sciences, University of Yaoundé I, Yaoundé, Cameroon; 4 Goulfey District Hospital, Goulfey, Cameroon

**Keywords:** Diabetes, Diabetic retinopathy, Photocoagulation, Cameroon

## Abstract

**Background:**

Diabetic retinopathy (DR) is the most common microvascular complication of diabetes. It can lead to significant visual loss. The aim of this study was to determine the frequency and clinical profile of diabetic retinopathy, and assess the outcomes of laser photocoagulation therapy in a diabetic population in Cameroon.

**Methods:**

We carried out a prospective cohort study during 24 months in the Department of Ophthalmology of the Douala General Hospital, Cameroon. We included all diabetic patients who were referred from diabetes clinics for ophthalmologic evaluation. Data included type and duration of diabetes, visual acuity, intra-ocular pressure, results of fundoscopy and fluorescein angiography, and outcomes two months after treatment with laser photocoagulation.

**Results:**

We included 407 patients; 88% had type 2 diabetes. Their mean duration of diabetes was 6.4 years (SD=6.6). Forty point three percent (164/407) of patients were found to have DR on fundoscopy. Of the 164 patients with DR, 63.4% (104/164) had non-proliferative and 36.6% (60/164) had proliferative DR. Diabetic maculopathy was found in 14.5% (59/407) of all participants, and 36% (59/164) of patients with DR. There was a strong correlation between the duration of diabetes and retinopathy status (p < 0.001, r = 0.9541). Overall, 17.4% (71/407) of patients were eligible for laser photocoagulation. Of these, 66.2% (47/71) were treated, and 78.7% (37/47) of treated patients came back for control two months later. Among these treated patients an improvement of the retinopathy was noted in 73% (27/37), no change in 16.2% (6/37) and a worsening in 10.8% (4/37). Severe proliferative DR was significantly associated with treatment failure (p < 0.001).

**Conclusions:**

The frequency of DR may be high among diabetic patients in Cameroon. There was a good uptake of laser photocoagulation therapy among patients affected by DR in our setting, with good treatment outcomes. Interventions to prevent diabetes and increase the precocity of diagnosis and treatment of DR should be scaled up.

## Background

Over the past century, diabetes mellitus was considered to be a rare medical condition. However, surveys of the global burden of disease indicate that non-communicable diseases will become the leading cause of mortality worldwide in 2030 [1]. Sub-Saharan African countries are currently facing an epidemiological transition with an increasing prevalence of diabetes alongside other non-communicable diseases [2]. In 2010, 12.1 million people were estimated to be living with diabetes in Africa, and this is projected to increase to 23.9 million by 2030 [3]. The prevalence of diabetes is 5.7% in urban Cameroon, with an estimated 1 million diabetic population, 70% of whom remain undiagnosed [4]. Microvascular complications of diabetes have been shown to be highly prevalent in African diabetic patients [5]. Diabetic retinopathy (DR) is the most common microvascular complication of diabetes [6]. Some previous studies have reported DR rates of about 37% in diabetics in Cameroon [7,8].

Visual impairment from diabetic retinopathy occurs through two mechanisms: retinal neovascularization; this is known as proliferative retinopathy and accounts for the majority of severe visual loss. In addition, retinal blood vessels can become permeable and cause swelling of the center of the retina, called diabetic macular oedema. Clinically significant macular oedema is a leading cause of moderate visual loss in diabetes. Proliferative retinopathy, severe non-proliferative retinopathy and clinically significant macular oedema can be considered as sight threatening retinopathy [6]. At these stages, unless it is treated, DR will almost inevitably evolve to irreversible blindness [9]. In facts, patients with untreated proliferative DR have over 50% chance of becoming blind within 5 years [10]. Prevention of retinopathy is the best approach in reducing the risk of blindness in diabetic patients. Laser photocoagulation for sight threatening diabetic retinopathy is not widely available in sub-Saharan Africa.

This study aimed at determining the frequency and severity of diabetic retinopathy, and assessing the outcome of laser photocoagulation therapy in a group of diabetic patients in Cameroon.

## Methods

### Ethical considerations

This study was performed in accordance with the guidelines of the Helsinki Declaration and was approved by the National Ethics Committee of Cameroon. We obtained written informed consent for all the participants.

### Study design and setting

We carried out a prospective cohort study from October 2004 through October 2006 in the Department of Ophthalmology of the Douala General Hospital, which has the best experience in laser photocoagulation in Cameroon. Douala is the administrative headquarter of the Littoral Region and the economic capital of the Republic of Cameroon. It is one of the two largest cities of Cameroon, with a population estimated at two million inhabitants [11].

### Study population and sampling

Participants were identified by convenience and consecutive sampling of all the diabetic patients seen during study the period in the Department of Ophthalmology of the Douala General Hospital, and consenting to participate. These diabetic patients were referred from diabetes clinics for ophthalmologic evaluation. We excluded patients with incomplete data.

### Data collection, variables and measurements

The requested information was: sociodemographic characteristics such as age, sex, residency, type and duration of diabetes, visual acuity, intra-ocular pressure, results of dilated fundus examination and fluorescein angiography, and laser photocoagulation therapy outcomes after two months.

Presenting and best-corrected visual acuity was measured from projection charts and classified according to the 1997 WHO classification of visual impairment and blindness [12]. Intra-ocular pressure was measured by applanation tonometry incorporated into the biomicroscope. The pupils were then dilated with midriatic drops, a combination of tropicamide 0.5% (Mydriaticum®) and phenyephrine HCL 10% (Neosynephrine®) instilled 3 times at 5 mins interval. Fundoscopy was performed 45 mins after the last drop.

Biomicroscopy was performed with a + 90 dioptres lens. Indirect ophthalmoscopy was performed as a supplement with a 3-mirror lens in patients with high risk of retinal detachment to visualize the peripheral retina. According to the Diabetic Retinopathy Disease Severity Scale of the American Academy of Ophthalmology, findings were classified as no retinopathy, non-proliferative retinopathy (mild, moderate, or severe), or proliferative retinopathy (mild, moderate, severe or complicated) [13]. Diabetic maculopathy was classified as absent, mild, moderate or severe. Eyes with suspect or evident retinopathy were eligible for a fluorescein angiography.

Fluorescein angiography was performed with a KOWA RC-XV2 angiograph, after an initial red free photograph of the fundi. Serial photographs were taken with blue light after injecting 5 ml of sodium fluorescein 10% into a good antebrachial vein, under strict asepsis. Eyes with severe non-proliferative retinopathy, any proliferative retinopathy, and sight threatening maculopathy were considered for laser photocoagulation therapy.

Photocoagulation therapy was performed with a semiconductor diode Laser, after a full mydriasis. Anaesthetic drops (Amethocaine 0.5%) were first instilled on the cornea. Artificial tears (Lacryvisc®) were applied on a 3-mirrored lens, and then carefully placed on the cornea. The power of the Laser beam ranged from 125 to 400 nW, with an exposure time that ranged from 0.1 to 0.2 s, and a diameter that ranged from 75 to 600 μm depending on the region being treated and the retinopathy status. The number of impacts ranged from 500 to 1900 per session. The eyes were treated per quadrant in pan photocoagulation. Focal therapy was used in specific cases. A short course of local anti-inflammatory was prescribed after each treatment session. Patients were reviewed at two months, and another angiography was done for control. Laser treatment outcome was evaluated and classified as: i) an improvement if there was a regression of the DR; ii) no change if there was a persistence of vessels or vitreous hemorrhage needing further treatment; iii) a worsening if there was an evolution to dense hemorrhage and fibrovascular membranes needing vitrectomy. We used a “per person” approach to classify diabetic retinopathy and maculopathy, meaning that a bilaterally affected patient was classified based on his most affected eye. In patients who received laser treatment for both eyes, we reported the best treatment outcome.

All exams and therapeutic interventions were done by experienced ophthalmologists.

### Data analysis

Data were coded, entered and analyzed using STATA version 8.2. We described continuous variables using mean and standard deviation, and categorical variables using their frequency and percentage. The chi-square test or its equivalent was used to compare categorical variables and a *p* value less than 0.05 was considered statistically significant.

## Results

A total of 407 patients were included. Of these, 58.2% (237/407) were male, giving a sex ratio of 1.4. Their ages ranged between 13 and 84 years, with a mean age of 54.2 (SD=11.2). Eighty eight percent of patients had type 2 diabetes (Table 1). The age of patients at diagnosis of diabetes ranged from 5 to 81 years, with a mean of 46.8 years (SD=11.5). The duration of diabetes ranged from 0 to 36 years, with a mean of 6.4 years (SD=6.6).

**Table 1:** Background characteristics of study population

**Characteristic**	**Frequency (%), N = 407 or Mean ± (standard deviation)**
**Age (years)**	
Overall	54.2 (11.2)
Female	55.7 (12.3)
Male	53.2 (10.3)
**Gender**	
Male	237 (58.2)
Female	170 (41.8)
**Type of diabetes**	
Type 1	49 (12)
Type 2	358 (88)
**Treatment of diabetes**	
Only diet	4 (1)
Oral drugs	320 (78.6)
Insulin	62 (15.2)
Insulin and oral drugs	4 (1)
Missing data	17 (4.2)
**Duration of diabetes (years)**	
Overall	6.4 (6.6)
Type 1 diabetes	7.9 (6.9)
Type 2 diabetes	6.5 (6.6)

**Figure 1:**
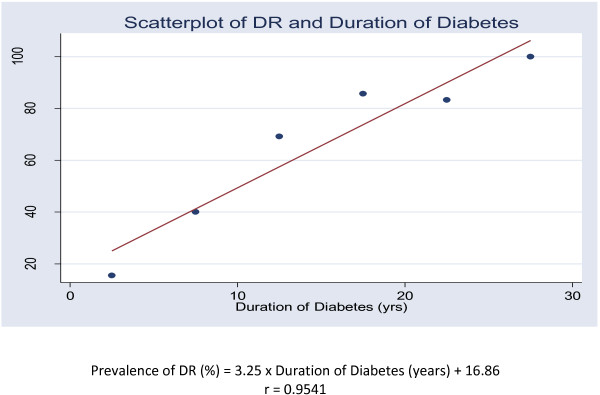
**Relation between the duration of diabetes and the prevalence of diabetic retinopathy.** Prevalence of DR (%) = 3.25 x Duration of Diabetes (years) + 16.86 r = 0.9541.

Forty point three percent (164/407) of patients had DR at various stages on fundoscopy (45.5% in type 1 and 38.8% in type 2). Of the 164 patients with DR, 63.4% (104/164) had non-proliferative and 36.6% (60/164) had proliferative DR. Fluorescein angiography was performed in 98.3% (400/407) of patients, giving a DR frequency of 42.2% (169/400). The frequency of retinopathy and its severity are summarized in Table 2. The frequency of sight threatening retinopathy (severe non-proliferative and proliferative) was 18.2%. Thirty seven (61.7%) patients of the 60 patients with proliferative DR presented complications including vitreoretinal haemorrage (51.7%; n = 31), retinal detachment (3.3%; n = 2) and neovascular glaucoma (6.7%; n = 4). Diabetic maculopathy was found in 14.5% (59/407) of all participants, 18.4% (9/49) of type 1 diabetic patients and 14% (50/358) of type 2 diabetic patients; and it was found in 36% (59/164) of patients with DR. Sixty-one percent (36/59) exhibited a mild maculopathy, 23.7% (14/59) a moderate maculopathy and 15.3% (9/59) a severe maculopathy. There was a strong correlation between the duration of diabetes and retinopathy status (p < 0.001, r = 0.95), Figure 1.

**Table 2:** Frequency and severity of diabetic retinopathy (DR) on fundoscopy and angiography

**Severity of DR**	**Fundoscopy, n (%)**	**Angiography, n (%)**
	**N = 407**	**N = 400**
**No DR**	243 (59,7)	231 (57,8)
**Non proliferative DR**	104 (25.6)	108 (27)
Mild	50 (12,3)	45 (11,2)
Moderate	43 (10,6)	51 (12,8)
Severe	11 (2,7)	12 (3)
**Proliferative DR**	60 (14.7)	61 (15,2)
Mild	7 (1,7)	7 (1,7)
Moderate	13 (3,2)	16 (4)
Severe	40 (9,8)	38 (9,5)

Overall, 17.4% (71/407) of patients had at least severe non proliferative DR and thus were eligible for laser photocoagulation. Of these, 66.2% (47/71) were treated, 10.6% (5/47) with severe non-proliferative and 89.4% (42/47) with proliferative retinopathy. Among patients with non-proliferative retinopathy, 10.6% (11/104) underwent laser photocoagulation therapy. Of the 60 patients with proliferative retinopathy, 70% (42/60) had photocoagulation therapy. Of the 47 patients who underwent photocoagulation treatment, 37 (78.7%) came back for control two months later. Among these treated patients an improvement of the retinopathy was noted in 73% (27/37), no change in 16.2% (6/37) and a worsening in 10.8% (4/37). Severe proliferative DR was significantly associated with treatment failure (p < 0.001).

## Discussion

The aim of this study was to determine the frequency and clinical profile of diabetic retinopathy, and assessing the outcomes of laser photocoagulation therapy in a diabetic population in Cameroon. Data will contribute in designing policies to reduce visual impairment and improve the quality of life of diabetic patients in Cameroon.

Using angiography, the frequency of diabetic retinopathy was 42.2% in our study population. In previous studies in Cameroon, Moukouri *et al.* in 1995 and Sobngwi *et al.* in 1999 found retinopathy prevalence of 37.3% and 37.5% respectively [7,8]. In agreement with these reports, our findings point out that retinopathy is frequent in diabetic patients in Cameroon. Similar prevalence rates have been reported in other settings: 40.30% in USA by Kempen *et al.* and 44% by Ben *et al.* in Tunisia [14,15]. In general, the prevalence of DR varies widely among populations, for example, from 17.9% in Ghana [16], to 60.78% in Senegal [17]. This variability could be explained by differences in sociocultural background (nutritional and behavioral habits including the level of physical activities). Much more, some differences in susceptibility to DR are thought to exist among different ethnic groups [6].

We found that the duration of diabetes strongly correlates with the severity of retinopathy (p < 0.001, r = 0.9541) in our study population. In facts, prolonged exposure to hyperglycaemia is an established risk factor of DR, highlighting the crucial need of an optimal control of blood sugar to prevent DR. This a very difficult task in developing countries like Cameroon that face a serious lack of health care facilities for diabetes management. Consequently, delayed diagnosis and ineffective management of diabetes occurring in these settings have a great impact in DR onset and progression. Much more, as microvascular complications and for instance DR are related to the duration of diabetes, early detection of retinopathy is an important preventive strategy [18]. Interestingly, type 2 diabetes, which is the most frequent type of diabetes, has an asymptomatic phase with actual diabetic hyperglycemia before clinical diagnosis. This phase has been estimated to last at least 4 to 7 years [19]. Therefore, identifying DR from newly diagnosed diabetes is valuable to the prevention and appropriate treatment of diabetic retinopathy in the early stage. Another known risk factor of DR is hypertension. Unfortunately we do not provide data on the implication of hypertension in our study. However, it has been shown that DR can progress despite optimal control of blood sugar and blood pressure [20].

Regarding the severity of DR, 57.8% of affected patients had non-proliferative DR on angiography, a finding in agreement with the 68.7% found by Nanfack *et al.* in Yaoundé, Cameroon [21]. The diabetic maculopathy rate was 14.5% in our study. This is about twofold the prevalence found by Nanfack *et al.* (7.15%) in Cameroon, Rema *et al.* (6.4%) in India and Khandekar *et al.* (6.3%) in Oman [21-23]. Seventeen point four percent of our patients had at least severe non-proliferative DR and thus required treatment by laser photocoagulation. Of these patients, 66.2% had a treatment. This is a good treatment uptake coverage compared to the 26.7% reported by Nanfack *et al.* in Yaoundé, Cameroon [21]. However, these findings emphasize the need of increasing the access to treatment for the patients affected by DR. In this way, the treatment outcomes in our study are encouraging: 73% of the patients who were treated and who had an angiography two months after treatment for control had an improvement. The association of severe proliferative DR with a poor treatment outcome in our study highlights the necessity of early diagnosis and treatment. The numerous cases of severe retinal disease found in our study is a call for the introduction vitreoretinal surgery in the routine ophthalmologic practice in Cameroon.

Our findings are limited by the fact that the study has been conducted in an urban context and the patients living in rural areas were not included, as well as diabetic patients with low socioeconomic background who cannot afford a good management of their disease and an ophthalmologic follow up. On the other hand, one of the strengths of our study is the accuracy of our data, since we diagnosed DR by both angiography and funduscopy which showed a very high diagnostic concordance.

## Conclusions

Our findings indicate that the frequency of DR may be high among diabetic patients in Cameroon. There was a good uptake of laser photocoagulation therapy among patients affected by DR in our setting, with good treatment outcomes. Yet, access to laser photocoagulation should be increased. It is of utmost importance to prevent the onset and the progression of DR in diabetic patients through optimal control of blood sugar and blood pressure. Acting upstream, it is crucial to scale up primary preventive interventions to break the increasing prevalence of diabetes in Cameroon as prevention is cost-effective.

## Competing interests

The authors declare that they have no competing interests.

## Authors’ contributions

AMJ conceived the study, collected and analyzed the data, and drafted the manuscript. JJNN contributed in data analysis and drafted the manuscript. AE and CEM contributed in study design, data collection and critically reviewed and revised the manuscript. JJRB critically reviewed and revised the manuscript. All authors approved the final version of the manuscript.

## Pre-publication history

The pre-publication history for this paper can be accessed here:

http://www.biomedcentral.com/1471-2415/14/19/prepub
